# What Is the Clinical Impact of Stress CMR After the ISCHEMIA Trial?

**DOI:** 10.3389/fcvm.2021.683434

**Published:** 2021-06-04

**Authors:** Théo Pezel, Luis Miguel Silva, Adriana Aparecia Bau, Adherbal Teixiera, Michael Jerosch-Herold, Otávio R. Coelho-Filho

**Affiliations:** ^1^Division of Cardiology, Department of Medicine, Johns Hopkins University, Baltimore, MD, United States; ^2^Department of Cardiology, Lariboisiere Hospital, University of Paris, Inserm, UMRS 942, Paris, France; ^3^Discipline of Cardiology, Faculty of Medical Science – State University of Campinas – UNICAMP, Campinas, São Paulo, Brazil; ^4^Noninvasive Cardiovascular Imaging Program and Department of Radiology, Brigham and Women's Hospital, Boston, MA, United States

**Keywords:** cardiovascular magnetic resonance, stress testing, myocardial ischemia, cardiovascular events, coronary revascularization, stable coronary disease

## Abstract

After progressively receding for decades, cardiovascular mortality due to coronary artery disease has recently increased, and the associated healthcare costs are projected to double by 2030. While the 2019 European Society of Cardiology guidelines for chronic coronary syndromes recommend non-invasive cardiac imaging for patients with suspected coronary artery disease, the impact of non-invasive imaging strategies to guide initial coronary revascularization and improve long-term outcomes is still under debate. Recently, the ISCHEMIA trial has highlighted the fundamental role of optimized medical therapy and the lack of overall benefit of early invasive strategies at a median follow-up of 3.2 years. However, sub-group analyses excluding procedural infarctions with longer follow-ups of up to 5 years have suggested that patients undergoing revascularization had better outcomes than those receiving medical therapy alone. A recent sub-study of ISCHEMIA in patients with heart failure or reduced left ventricular ejection fraction (LVEF <45%) indicated that revascularization improved clinical outcomes compared to medical therapy alone. Furthermore, other large observational studies have suggested a favorable prognostic impact of coronary revascularization in patients with severe inducible ischemia assessed by stress cardiovascular magnetic resonance (CMR). Indeed, some data suggest that stress CMR-guided revascularization assessing the extent of the ischemia could be useful in identifying patients who would most benefit from invasive procedures such as myocardial revascularization. Interestingly, the MR-INFORM trial has recently shown that a first-line stress CMR-based non-invasive assessment was non-inferior in terms of outcomes, with a lower incidence of coronary revascularization compared to an initial invasive approach guided by fractional flow reserve in patients with stable angina. In the present review, we will discuss the current state-of-the-art data on the prognostic value of stress CMR assessment of myocardial ischemia in light of the ISCHEMIA trial results, highlighting meaningful sub-analyses, and still unanswered opportunities of this pivotal study. We will also review the available evidence for the potential clinical application of quantifying the extent of ischemia to stratify cardiovascular risk and to best guide invasive and non-invasive treatment strategies.

## Introduction

After progressively dropping for decades, cardiovascular (CV) mortality due to coronary artery disease (CAD) has recently increased, and the associated healthcare costs are projected to double by 2030 (1). Although the current European and American guidelines for chronic coronary syndromes recommend non-invasive cardiac imaging for patients with suspected CAD (2, 3), the impact of non-invasive imaging strategies to guide initial coronary revascularization and improve long-term outcomes is still under debate (4). Indeed, the International Study of Comparative Health Effectiveness with Medical and Invasive Approaches (ISCHEMIA) trial has recently shown the lack of benefit to an initial revascularization strategy as compared to optimal medical therapy (5).

Cardiovascular magnetic resonance (CMR) imaging is an accurate technique to assess ventricular function, the extent of myocardial scar and viability, and inducible myocardial ischemia (6–9). Furthermore, the diagnostic accuracy (10–14), cost-effectiveness (15), and prognostic value (8, 9, 16, 17) of stress CMR compare favorably to other functional non-invasive tests, such as nuclear perfusion or stress echocardiography. A recent study has even demonstrated (18) that a first-line stress CMR-based non-invasive strategy was non-inferior in terms of outcomes, with a lower incidence of coronary revascularization, compared to an initial invasive approach guided by fractional flow reserve (FFR) in patients with stable angina. Consistently, several studies have underlined the high negative predictive value of stress CMR to detect CAD (6–9). Therefore, it can be hypothesized that myocardial ischemia detected by stress CMR could be helpful in guiding coronary revascularization and optimizing the management of these patients (19).

In the present review, we will discuss the current state-of-the-art data on the prognostic value of stress CMR assessment of myocardial ischemia in light of the ISCHEMIA trial results, highlighting some sub-analyses and still unanswered questions of this pivotal study. We will also review the available evidence for the potential clinical application of assessing the extent of ischemia to stratify CV risk and to best guide invasive and non-invasive treatment strategies.

## Lessons From the Ischemia Trial

Before the ISCHEMIA trial, the BARI 2D (Bypass Angioplasty Revascularization Investigation 2 Diabetes) (20) and COURAGE (Clinical Outcomes Utilizing Revascularization and Aggressive Drug Evaluation) (21) trials failed to demonstrate any significant benefit from coronary revascularization compared to medical treatment in the occurrence of all-cause death or CV outcomes in patients with angiographic evidence of obstructive CAD. The ISCHEMIA trial is to-date the largest well-designed trial comparing an invasive strategy to optimal medical therapy in patients, 5,179 in total, with moderate or severe ischemia on stress tests. The presence of at least moderate ischemia on stress tests was defined as follow: (i) ≥5% myocardium ischemic for nuclear perfusion; (ii) ≥2/16 segments with stress-induced severe hypokinesis or akinesis for echocardiography; (iii) ≥12% myocardium ischemic, and/or wall motion ≥3/16 segments with stress-induced severe hypokinesis or akinesis for CMR; and (iv) as compared to the baseline ECG tracing, additional exercise-induced horizontal or downsloping ST- segment depression ≥1.5 mm in 2 leads or ≥2.0 mm in any lead; ST-segment elevation ≥1 mm in a non-infarct territory for exercise test without imaging (5). Among the 5,179 patients randomized, 45% had a moderate ischemia defined by an extent of ischemia <10%. Therefore, ISCHEMIA trial was a mix of patients with moderate or severe ischemia which limits the extrapolation of these data. However, some larges studies suggest that the threshold of ≥10% ischemic myocardium could be the exact threshold to define revascularisation benefit (22). Notably, more severe ischemia was diagnosed with exercise test in 25% of patients.

**Figure 1:**
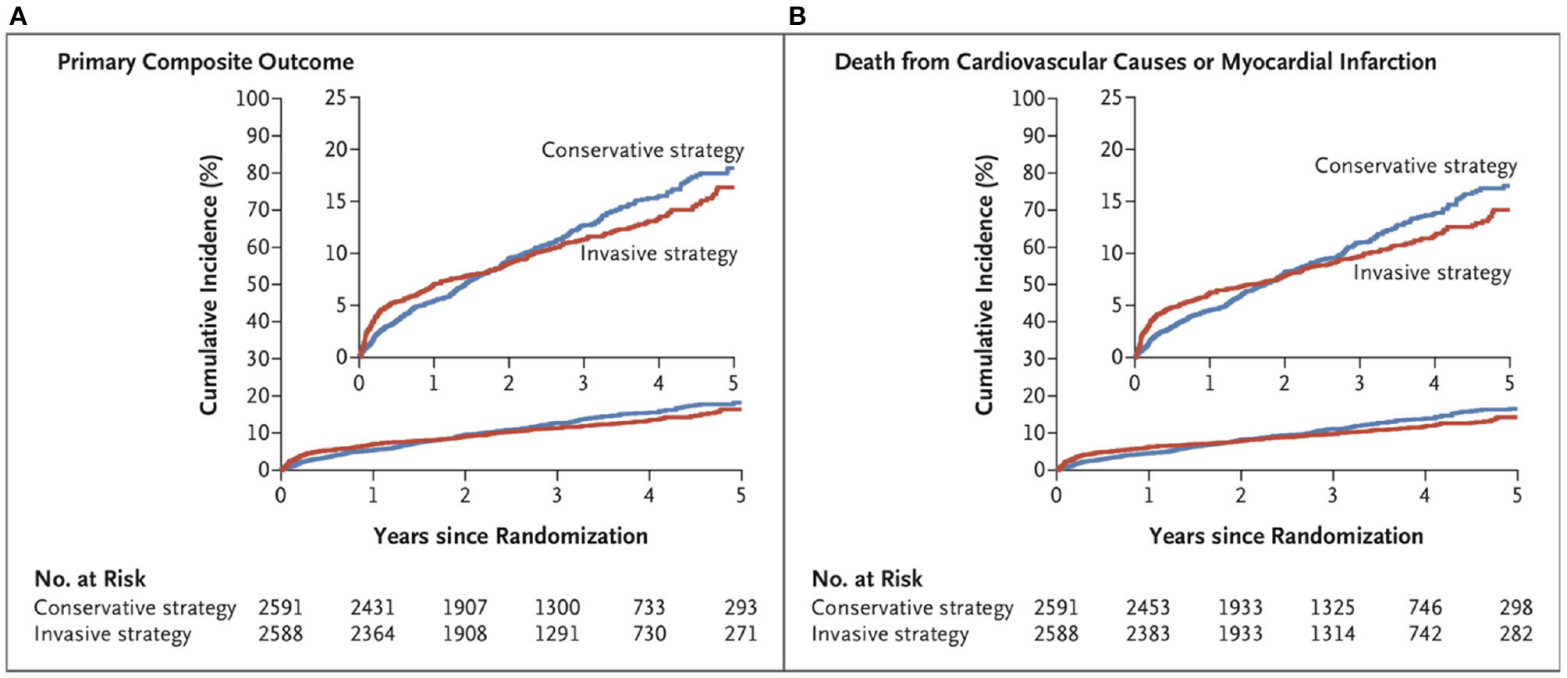
Cumulative incidence curves for the primary composite outcome and other outcomes, from ISCHEMIA trial (5). **(A)** shows the cumulative incidence of the primary composite outcome of death from cardiovascular causes, myocardial infarction, or hospitalization for unstable angina, heart failure, or resuscitated cardiac arrest in the conservative-strategy group and the invasive- strategy group. **(B)** shows the cumulative incidence of death from cardiovascular causes or myocardial infarction.

This study highlighted the crucial role of optimized medical therapy and the lack of benefit to an initial invasive strategy (5) (Figure 1). This trial had several strengths. First, it was a randomized clinical trial with a rigorous design requiring the documentation of obstructive CAD evaluated on coronary computed tomography angiography (CCTA) prior to randomization assessed by an independent core laboratory. Moreover, this trial was not industry funded, and the rate of patients lost to follow-up was very low (<1%). The primary outcome was a composite of cardiovascular death, myocardial infarction (MI), or hospitalization for unstable angina, heart failure, or resuscitated cardiac arrest. Beyond the usual CV outcomes, the study performed a rigorous evaluation of quality-of-life measures. Finally, control of CV risk factors was optimal in the entire cohort, based on systolic blood pressure and LDL cholesterol levels. Consistently, follow-up appeared to be very good with excellent adherence to medical treatment in both groups (about 80% at the end of follow-up) (5).

Despite these important strengths, some aspects warrant further attention. While the ISCHEMIA trial, which included procedural infarctions, showed the lack of benefit of initial coronary revascularization at a median follow-up of 3.2 years, a sub-analysis that excluded procedural infarctions suggested that initial coronary revascularization could improve outcomes at 5 years of follow-up (5). Indeed, during the first 6 months of follow-up, the estimated cumulative CV events rate was 5.3% in the invasive group and 3.4% in the medical treatment group (difference: 1.9%; 95% CI: 0.8–3.0). However, at 5 years, the cumulative event rate was 16.4% in the invasive group and 18.2% in the medical treatment group (difference, −1.8%; 95% CI, −4.7 to −1.0). This result suggests that initial coronary revascularization could improve outcomes at 5 years of follow-up. A similar finding was described for the composite outcome including CV death or MI and angina-related quality of life with a cumulative event rate of 14.2% in the invasive group and 16.5% in the medical treatment group (difference: −2.3%; 95% CI: −5.0 to −0.4) (5). This is consistent with another secondary analysis suggesting greater improvement in health status scores for patients in the invasive group compared to patients in the medical treatment group. Moreover, previous large observational cohort studies also suggested a clinical benefit to early coronary revascularization in patients with inducible ischemia at a mean follow-up of 4.6–5.5 years (23, 24). Therefore, the extended follow-up of the ISCHEMIA trial will provide more information since survival curves have crossed through the study period.

Regarding the presence of symptoms, 35% of participants had no angina, 44% had angina <3 times a month, and only 20% had daily or weekly angina. In addition, the Seattle Angina Questionnaire score was 73 ± 19 in the invasive strategy group and 75 ± 19 in the medical treatment group (5). This indicates that the majority of participants in both groups were asymptomatic or only mildly symptomatic at baseline. Therefore, these findings suggest that the optimal medical treatment in asymptomatic or mildly symptomatic patients may be the best initial strategy without the benefit of coronary revascularization. However, in symptomatic patients with frequent angina episodes, and a fortiori in cases of severe ischemia, an invasive strategy would be a reasonable complementary approach to the optimal medical treatment for effective angina relief, in line with the current guidelines (2, 3). Therefore, one major message of the ISCHEMIA trial was the excellent capability of coronary revascularisation to relief symptoms. In addition, all patients with left main stenosis of at least 50% were excluded from the ISCHEMIA trial, although left main stenosis is the most severe CAD involvement in terms of ischemia extent and risk of cardiovascular events (5). However, these patients would likely benefit most from coronary revascularization. Furthermore, because coronary angiography was performed before the randomization, patients with coronary anatomy that might be associated with a very high risk for adverse outcomes were likely not randomized but sent directly to invasive revascularization. Although 54.8% of patients had severe ischemia, 45.2% had mild to moderate ischemia after core laboratory analysis (5). Notably, the ISCHEMIA trial was not designed for assessing the clinical value of ischemia testing because there was no control group without ischemia testing or with a negative stress test.

## Diagnostic Performance of Stress CMR Compared to Other Methods

**Figure 2:**
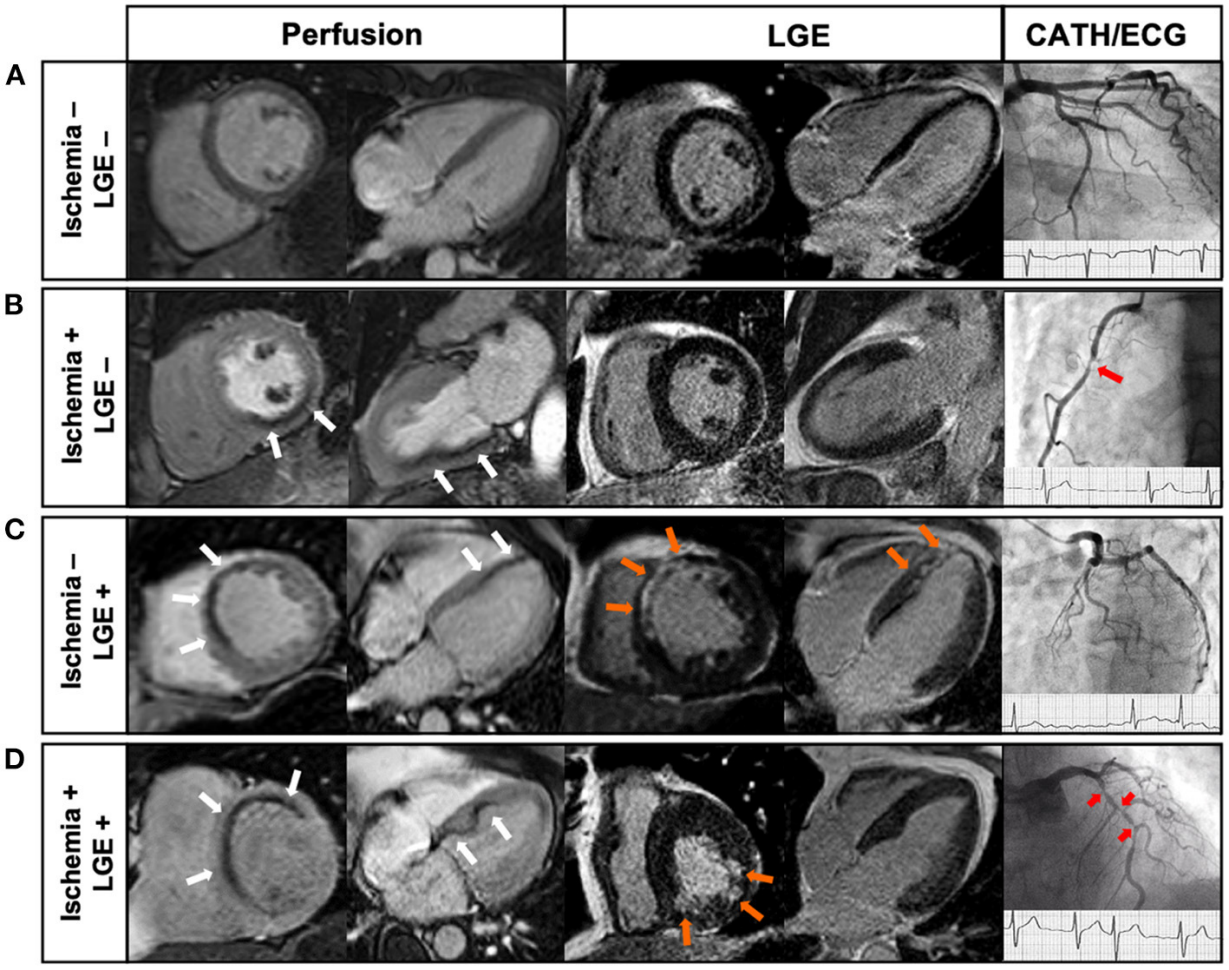
Examples of Clinical cases of stress CMR (26). **(A)** 68-year old male with atypical chest pain. Stress CMR revealed no perfusion defect and LGE was negative, ruling out the diagnosis of CAD. **(B)** 71-year old male with dyspnea on exertion. First-pass myocardial stress perfusion images revealed a reversible perfusion defect of the inferior wall (3 segments) (*white arrows*) without LGE, indicative of myocardial ischemia suggestive of significant RCA stenosis, confirmed by coronary angiography (*red arrow*). **(C)** 62-year old female with prior anterior STEMI treated by PCI 4 years before, referred for atypical chest pain. CMR showed a subendocardial anteroseptal scar on LGE (*orange arrows*), with a colocalization of the perfusion defect (*white arrows*), and therefore no inducible ischemia. Coronary angiography confirmed the absence of significant stenosis. **(D)** 69-year old male with AF and a history of inferior NSTEMI treated by PCI 8 years before, presenting with dyspnea on exertion. CMR showed a subendocardial scar on the inferior wall on LGE sequences (*orange arrows*), and a perfusion defect of the antero-septo-basal wall (4 segments) (*white arrows*) on first-pass perfusion images, indicative of inducible myocardial ischemia. Coronary angiography showed several high-grade stenoses of the LAD (*red arrows*). CAD, coronary artery disease; CMR, cardiac magnetic resonance; LAD, left anterior descending; LGE, late gadolinium enhancement; MI, myocardial infarction; NSTEMI, non ST-segment elevation myocardial infarction; PCI, percutaneous coronary intervention; RCA, right coronary artery; STEMI, ST-segment elevation myocardial infarction.

Although the American guidelines published in 2012 advise using stress CMR to detect obstructive CAD with a class II recommendation (level of evidence B), while other imaging methods had a class I recommendation (3), stress CMR has recently been added as a class I imaging technique for chronic coronary syndromes assessment (level B of evidence) in the current European guidelines, published in 2019 (2) (Table 1). Indeed, in these guidelines, stress CMR is recommended to guide coronary revascularization and to stratify symptomatic patients with intermediate risk of CAD (I, B), alongside other stress imaging approaches (2). Interestingly, these guidelines advocate quantitative perfusion CMR as a means of helping to identify patients with coronary microvascular disease, assigning it the same class of recommendation and level of evidence as PET (IIb, B). The adaptation of these European guidelines took into account recent data from a compilation of 26 studies, including more than 11,000 patients, a predicted sensitivity of 89%, and a specificity of 80% in the detection of CAD by stress CMR (11). A very recent meta-analysis has shown the superiority of stress CMR regarding the diagnostic test accuracy for detecting obstructive CAD compared to dobutamine stress echocardiography (25). Some clinical cases of stress CMR are illustrated in Figure 2.

**Table 1:** Comparison between European and American guidelines regarding the use of stress CMR.

	**Recommendation for the use of stress CMR**	**Class (Level)**
**European Guidelines** Knuuti et al. (2) ESC Guidelines for the diagnosis and management of chronic coronary syndromes.	**All non-invasive functional stress testing** including Stress echocardiography, SPECT and Stress CMR, are recommended as the initial test to diagnose CAD in symptomatic patients.	**I** (B)
**American Guidelines** Fihn et al. (3)	**Pharmacological stress with CMR** can be useful for patients with an intermediate to high pretest probability of obstructive CAD.	**II** (B)
ACCF/AHA/ACP/AATS/PCNA/SCAI/STS guideline for the diagnosis and management of patients with stable ischemic heart diseases.	Exercise stress with **nuclear imaging** or **echocardiography** is recommended for patients with an intermediate to high pretest probability of obstructive CAD.	**I** (B)

Regarding single photon emission computed tomography (SPECT), the MR-IMPACT II trial has demonstrated non-inferior performance of stress CMR in the presence of at least one diseased vessel and superior performance in multi-vessel disease (14). This is consistent with the CE-MARC study, which also reported the superiority of stress CMR in single-vessel disease compared to SPECT related to the higher spatial resolution of CMR than SPECT. Moreover, it seems those with multi-vessel disease stand to benefit the most from CAD diagnosis by CMR due to the better spatial resolution (12, 27), and particularly using quantitative stress CMR imaging (28). Recent meta-analyses (10, 11) comparing all non-invasive stress test methods to FFR confirmed the diagnostic accuracy of stress CMR compared to other methods. The MR IMPACT II trial (*n* = 533) showed stress CMR is a good and efficient alternative to SPECT with greater sensitivity (0.67 vs. 0.59, *p* = 0.024), but lower specificity (0.61 vs. 0.72, *p* = 0.038) (14). The CE-MARC study (*n* = 752), on the other hand, demonstrated greater sensitivity (87 vs. 67%, *p* < 0.0001) and negative predictive value (91 vs. 79%, *p* < 0.0001) for CMR vs. SPECT, while specificity (83% vs. 83%, *p* = 0.916) and positive predictive value (77 vs. 71%, *p* = 0.061) were similar (12). In a sex-specific analysis from the CE-MARC study, CMR had greater sensitivity in women and men (89 vs. 86%, *p* = 0.57) than SPECT (51 vs. 71%, *p* = 0.007) in identifying coronary angiography significant stenosis without a significant difference between sexes (27).

Beyond the traditional non-invasive stress-test methods, computed tomography with fractional flow reserve (FFR-CT) is a new non-invasive technique that has developed significantly in recent years (29). Recently, a study compared the diagnostic performance of FFR-CT and stress perfusion CMR in 110 patients with stable chest pain referred to invasive coronary angiography (30). Interestingly, both methods presented similar overall diagnostic accuracy. Sensitivity for prediction of obstructive CAD was highest for FFR-CT (97%), whereas specificity was highest for stress CMR (88%).

## Prognostic Value of Stress CMR

The long-term prognostic value of stress CMR is well-established in large studies (8, 9, 16, 17). In the Euro-CMR registry (27,000 consecutive CMR studies in 15 European countries), 1,706 patients with suspected CAD presenting with a normal stress CMR had a low CV event rate (1%/year) (31). Another large multicenter study, assessing 9,151 patients with a median follow-up of 5 years, showed that stress CMR is independently associated with all-cause death (9). While the annual death rate of patients with a normal stress CMR in this study was 1.4% per year, it increased to 4.0% per year in patients with an abnormal stress CMR. Moreover, a meta-analysis (19 studies, 11,636 patients, mean follow-up of 2.7 years) supported the excellent negative prognostic value of stress CMR, describing an annualized rate of CV outcomes of 4.9% per year for patients with an abnormal stress CMR vs. only 0.8% per year for a normal stress CMR (16). A more recent meta-analysis (165 studies, 122,721 patients, mean follow-up of 2.7 years) studied all non-invasive cardiac modalities to detect myocardial ischemia and demonstrated that the annual event rates for CV death and non-fatal MI have been consistently reported as <1% for patients with a normal stress CMR (32). Regarding sex difference, Coelho-Filho et al. showed that stress CMR myocardial perfusion imaging is an effective and robust risk-stratifying tool for patients of either sex presenting with ischemia (33). However, among individuals with a negative stress CMR, some data demonstrate lower rates of CV events in women than in men, with annualized CV event rates of 0.3% in women vs. 1.1% in men (33).

Recently, the SPINS (Stress CMR Perfusion Imaging in the United States) study investigated the prognostic value of stress CMR in the largest CMR retrospective cohort of patients with stable chest pain in the US (8). In this study, patients with intermediate pre-test probability of CAD who had both negative ischemia and late gadolinium enhancement (LGE) (67% of the patients) experienced a low annualized rate of CV death or non-fatal myocardial infarction (0.6%) (8). On the other hand, patients with both positive ischemia and LGE had an annual event rate of 4.5% per year. In addition, several recent studies have shown that the prognostic value of stress CMR was also observed in subgroups challenging to evaluate using other non-invasive methods, such as patients with obesity (26, 34), prior CABG, or (35) atrial fibrillation during stress testing (36, 37), and very elderly individuals (38). Beyond the presence of ischemia, a sub-study of SPINS has recently shown that the presence of unrecognized or recognized MI portended an equally significant risk for CV events, even after adjustment for the presence of ischemia (37). These findings highlight the importance of using both ischemia and myocardial scar detected by stress CMR to stratify the risk of CV events.

In addition, several studies have emphasized that the extent of inducible ischemia assessing by the number of ischemic segments was a strong and independent predictor of MACE and CV mortality (39, 40), in both patient without (41) or with known CAD (42).

Beyond the prognostic value, it could be worthwhile to assess the incremental prognostic value of the stress CMR over traditional risk factors or comorbidities. In a cohort of 513 patients, Jahnke et al. described an incremental prognostic value of stress CMR, either by perfusion or by wall motion, over traditional factors such as age, sex, smoking, and diabetes, in predicting CV death and non-fatal myocardial infarction (43). Moreover, another study assessing 815 consecutive patients referred for CAD detection demonstrated that stress CMR results in a better reclassification to predict CV outcomes beyond traditional risk factors, specifically in patients at moderate to high pre-test clinical risk and in patients with previous CAD (44). All these findings are in agreement with myocardial perfusion SPECT or echocardiographic studies, which have shown the incremental prognostic value of ischemia in predicting CV mortality (45, 46).

## Subgroups With Specific Benefit of Revascularisation: Heart Failure Patients

Coronary artery disease is the main risk factor for heart failure and accounts for more than two-thirds of heart failure cases with reduced left ventricular ejection fraction (LVEF) (47). Knowing that myocardial ischemia may represent a treatable cause of LV dysfunction (48), current guidelines recommend invasive or non-invasive assessment for obstructive CAD in all newly diagnosed heart failure cases (49, 50). Indeed, coronary revascularization in patients with ischemic cardiomyopathy and reduced LVEF may improve LV dysfunction by reducing ischemia in a viable hibernating myocardium (2, 49, 50). Interestingly, a large multicenter registry has shown that the presence of inducible ischemia assessed by stress CMR was an independent predictor of all-cause mortality in patients with LVEF <55% (adjusted hazard ratio 1.8, *p* < 0.001) (9). A more recent stress CMR study has suggested that both the presence and extent of inducible ischemia were independent and strong predictors of a higher incidence of CV outcomes in a cohort of 1,053 patients with heart failure and LVEF < 40% (51).

**Figure 3:**
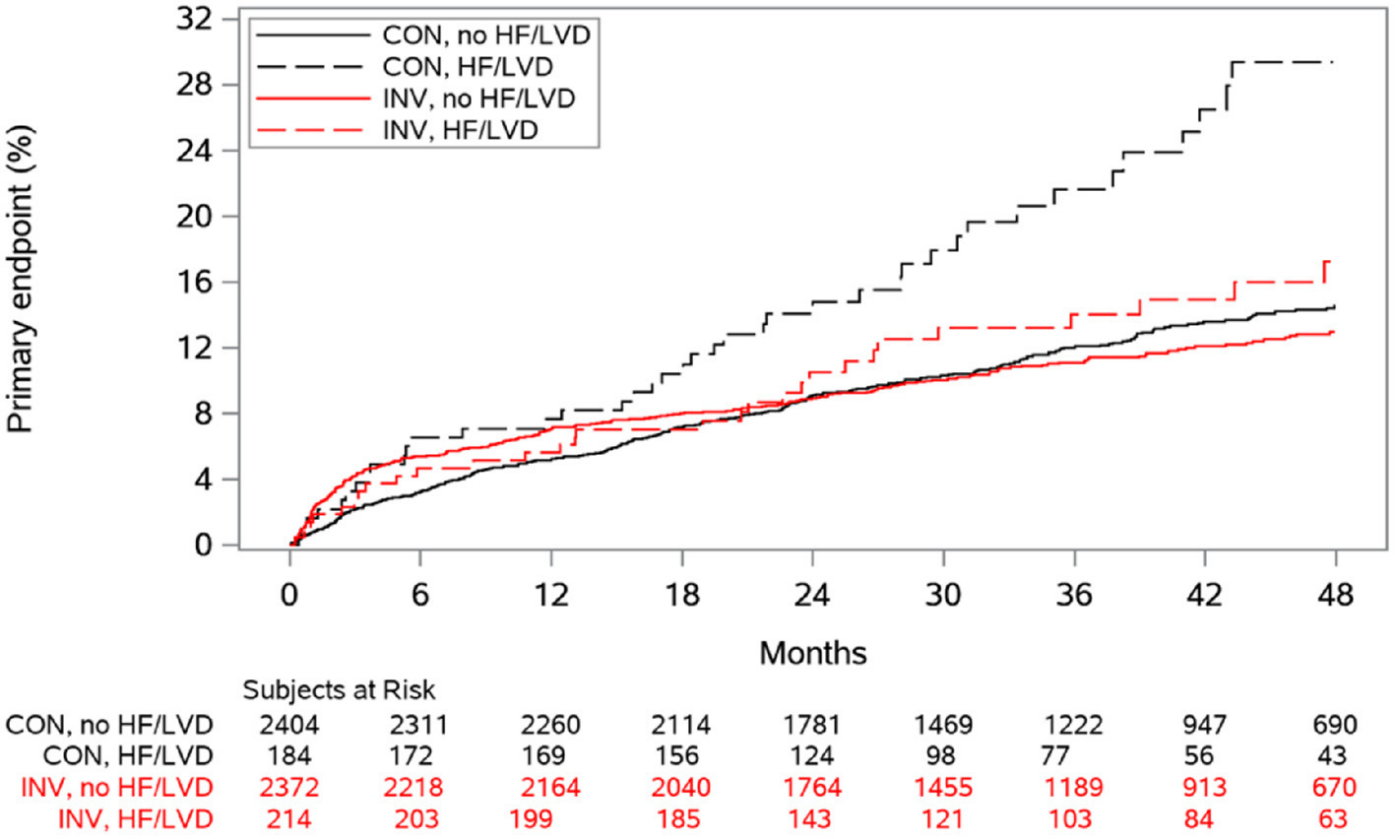
Cumulative incidence curves for the primary composite outcome according to randomized treatment and history of heart failure (HF) or left ventricular dysfunction (LVD), from ISCHEMIA trial (52). CON indicates conservative strategy; and INV, invasive strategy.

Although the ISCHEMIA trial has described the overall lack of benefit to early revascularization, it was not designed to investigate the population of patients with reduced LVEF. Indeed, the large majority of patients had LVEF ≥ 50% (median [IQR] = 60 [55–65]%) (5). Interestingly, a recent ancillary study of ISCHEMIA assessing only the subgroup of patients with LVEF 35–45% suggested a better event-free survival rate after an initial invasive strategy (52) (Figure 3). However, initial studies assessing the potential interest of coronary revascularization in patients with reduced LVEF did not suggest any benefit in terms of CV outcomes. For example, the results of the randomized STICH (comparison of surgical and medical treatment for congestive HF and CAD) and HEART (HF revascularization trial) studies, evaluating the prognostic value of coronary artery bypass graft (CABG) in patients with reduced LVEF, were negative at 5 years (48, 53).

However, the extended follow-up of STICH (median 9.8 years) has recently shown that surgical revascularization in addition to medical therapy resulted in a significant benefit for all-cause mortality and CV outcomes (54). Moreover, some non-randomized studies have also demonstrated the potential benefit of percutaneous coronary intervention (PCI) compared to medical treatment alone in patients with reduced LVEF (55). Therefore, all of these studies seem to show a real benefit to coronary revascularization in patients with both ischemia and reduced LVEF.

## Subgroups With Specific Benefit of Revascularisation: Patients With Severe Ischemia

Assessment of ischemia extent by stress CMR was previously described as a strong and independent prognostic factor in many cohort studies (39, 40). A prospective stress CMR study assessing 1,024 consecutive patients with suspected CAD suggested that simple quantification of the number of ischemic segments provides a good prognostic value to stratify the CV risk of patients (39). Indeed, this study suggested that patients with ≥1.5 ischemic segments presented a worse prognosis with a higher incidence of CV death, non-fatal myocardial infarction, or late coronary revascularization. More recently, Marcos-Garces et al. have shown that an extensive ischemic burden, assessed by number of ischemic segments using stress CMR, was related to a higher risk of long-term, all-cause mortality after a median follow-up of 6 years in a cohort of 6,389 consecutive patients with suspected CAD (40). Furthermore, the authors demonstrated that the long-term risk of all-cause mortality increased in parallel with the extent of ischemia, with a risk of death at 6 years of 8% in patients with <2 ischemic segments vs. 27% in those with a large ischemic burden, defined as >9 ischemic segments. Coronary revascularization was associated with a protective effect only in the restricted subset of patients with extensive CMR-related ischemia, defined as >5 ischemic segments. Moreover, the extension of myocardial ischemic burden using SPECT was also described as stratifying all-cause mortality in a large cohort of patients with suspected CAD. Indeed, this study demonstrated both short- and long-term survival benefits associated with revascularization in patients with significant (>10%) ischemic myocardium (56, 57). All these findings are in line with a previous functional imaging sub-study of the COURAGE trial. In the subset of patients who underwent serial functional testing with scintigraphy, PCI with a treatment target of ≥5% ischemia reduction resulted in improved outcomes and a greater reduction in ischemia compared with medical therapy alone (58).

Therefore, all of these studies suggest that there is a potential benefit to coronary revascularization for severe ischemia, i.e., an ischemia of more than 5 ischemic segments. However, the ISCHEMIA trial assessed only 54.8% of patients had severe ischemia after core laboratory analysis (5). One could thus imagine an interest in a new randomized controlled trial assessing the benefit of coronary revascularization, but only in patients with severe ischemia.

## The Potential Interest of Quantitative Perfusion CMR

Nuclear imaging with SPECT is most commonly used for clinical myocardial perfusion imaging, whereas PET is the gold-standard for the quantification of myocardial perfusion (59). More recently, technical improvements to the quantification of pathophysiological parameters of myocardial ischaemia in the CMR field allow to assess the myocardial perfusion using stress CMR without any exposure to ionizing radiation (59). Currently, the analysis of stress CMR perfusion scans is overwhelmingly based on a visual, observer-dependent assessment of contrast enhancement. Thus, an accurate and reproducible quantification of the burden of ischemia, such as quantification of myocardial blood flow (MBF) by CMR, may be useful in improving the assessment of the optimal medical therapy. Quantitative perfusion analysis provides incremental prognostic value over semiquantitative and qualitative data analysis, with an area under the receiver operating characteristic curve (AUC) of 0.85 vs. 0.75 (59, 60). MBF quantification techniques have been validated against coronary sinus flow (61) and PET MBF in healthy volunteers (62). Interestingly, there are different models for quantification of MBF including: tracer-kinetic modeling using blood-tissue exchange models (63), Fermi deconvolution analysis (64), and model-independent analysis (65). However, absolute measures remain variable with these techniques because they are tightly connected to the CMR sequence, and a lack of standardization exists between systems (59). Although not currently part of clinical practice, MBF quantification could allow for identification of multi-vessel coronary disease (28) and give a very accurate assessment of the extent of the ischemia, and not just the detection of microvascular disease, as previously mentioned (2).

Beyond obstructive CAD, invasive coronary flow reserve (CFR) or FFR evaluation emphasize the importance of detecting microvascular dysfunction. Indeed, a recent randomized controlled trial showed that in patients without obstructive CAD, personalized treatment guided by the results of CFR reduced anginal symptoms compared to conventional medical treatment (66). Current European guidelines suggest that CFR and/or microcirculatory resistance measurements should be considered in patients with persistent symptoms, but coronary arteries that are either angiographically normal or have moderate stenoses with preserved FFR (level IIa) (2). Notably, several studies have shown the excellent correlation between quantitative perfusion CMR and the diagnosis of microvascular dysfunction using invasive measurement (67). Indeed, microvascular disease may appear as a subendocardial concentric perfusion defect. Because this perfusion defect may not respect coronary territories, its diagnosis could be difficult. Quantification of MBF by CMR can be useful in such cases. Therefore, we could imagine a role for quantitative perfusion CMR to perform large therapeutic randomized controlled trials in this population, for which no treatment is recommended.

## Clinical and Cost-Effectiveness Impact of Stress CMR-Related Coronary Revascularization

Beyond diagnostic performance and prognostic value in patients with suspected CAD, a randomized controlled trial—the MR-INFORM study—has recently demonstrated that a diagnostic strategy based on stress CMR was non-inferior in terms of incidence of death, non-fatal myocardial infarction, or target-vessel revascularization compared to an invasive strategy with fractional flow reserve but with a lower use of coronary revascularization (18). Indeed, despite a similar pre-test probability of CAD of 75% in both groups, only 36% of patients who underwent invasive angiography in the stress CMR group required index coronary revascularization, as opposed to 45% in the FFR group (18).

Beyond the potential benefit of coronary revascularization, some recent studies have shown promising new therapy strategies targeting coagulation (68) and inflammation (69, 70) to decrease the risk CV outcomes in patients with CAD. However, these new therapies are associated with some side effects, such as an increased risk of bleeding and a risk of infection. Thus, this is crucial to be able to identify accurately the patients who will benefit most from these treatments in terms of the benefit/risk balance. Based on the studies showing an incremental prognostic value of stress CMR above traditional risk factors (8, 9, 43, 44), we can assume that an improved risk stratification using stress CMR could allow for the identification of high-risk patients who could benefit from treatment intensification, new therapy and/or revascularization.

Based on published average national payment rates from the Medicare Hospital Outpatient Prospective Payment System (71), the cost of stress CMR is usually lower than that of SPECT techniques and only slightly higher than stress echo, which makes it a cost-effective approach owing to its complementary diagnostic capabilities. Indeed, the SPINS study has shown that patients without ischemia or LGE experienced a very low incidence of CV events, little need for coronary revascularization, and low financial expenditure on subsequent ischemia testing in the US (8). Moreover, the lower cost of stress CMR compared to nuclear stress techniques or initial coronary angiography has recently been confirmed in a dedicated cost-effectiveness report from the SPINS study (15). Hypothetically, combining data from the public health systems of Europe (Germany, the UK, and Switzerland) and the US, the stress CMR approach—as opposed to coronary angiography as a single test—could result in a cost savings of up to 51% (72). All of these findings suggest that stress CMR could be helpful in reducing the costs of downstream testing, mainly due to a high negative predictive value. Therefore, stress CMR emerges as a highly attractive method for non-invasive risk stratification and further referral of high-risk patients.

## Future Directions After the Ichemia Trial

Although ischemia trial underlined the fundamental role of optimized medical therapy, the invasive approach clearly has benefits. Invasive therapy reduces symptomatic angina, with greater advantage in more symptomatic patients. It also reduces late MI and hospitalizations for unstable angina in ISCHEMIA trial (5). Indeed, while including procedural infarctions, the ISCHEMIA trial showed the lack of benefit of revascularisation, a sub-analysis that excluded procedural infarctions suggested better outcome in the invasive strategy group (5). In addition, another sub-analysis of ISCHEMIA at 5-year follow-up suggested that coronary revascularization could be beneficial in the subgroup of patients with inducible ischemia. Therefore, a longer-term follow-up of ISCHEMIA is important to understand these late benefits and early risks more fully. Moreover, approximately 8% of patients screened were found to have significant left main disease and were not randomized in ISCHEMIA trial. However, patients with left main disease have historically greater risks of cardiovascular events than other subgroups and theoretically derive greater benefits from coronary revascularization. Thus, for these patients, invasive management remains recommended (2). Moreover, a role for quantitative stress CMR can be hypothesized to accurately assess invasive approaches and then propose new prognostic stratification tools after an invasive approach has been performed. This review detailed the good results of stress CMR compared to other ischemia assessment methods in terms of diagnostic performance (10, 11), prognostic value (8, 16), and clinical impact compared to an invasive FFR strategy (18). However, among the 5,176 patients included in ISCHEMIA trial, stress CMR was performed in only 257 patients (5%) whereas the myocardial SPECT was carried out in 2,567 patients (49.6%). Knowing the superiority of stress CMR compared to SPECT (12), the ISCHEMIA trial probably does not accurately assess the prognostic value of stress CMR-based coronary revascularization guided by myocardial ischemia. Notably, the initial inclusion criterion for SPECT, which was the extent of the myocardial ischemia >10%, was modified during the study to >5% of the ischemic myocardium. Knowing the rather low spatial resolution of SPECT, a threshold of only 5% of the ischemic myocardium does not allow to identify accurately severe ischemia. Thus, one may wonder about the results of a new randomized controlled trial evaluating the interest of revascularization, in line with the design of the ISCHEMIA trial, but including patients with inducible ischemia defined only by stress CMR. Interestingly, a recent study assessing the external applicability of the ISCHEMIA trial has shown that only 4% of patients from a large registry fulfilled ISCHEMIA inclusion criteria (73), which suggests a very limited applicability of these findings to other patient cohorts.

## Safety and Limitations of Stress CMR

The excellent safety profile of stress CMR was demonstrated in a large registry of 11,984 patients using dipyridamole or dobutamine (74) and in the EuroCMR registry assessing 10,228 patients referred for stress CMR (31). The incidence of severe complications and non-severe complications was low, at 0.08 and 1.5%, respectively (74) and 0.07 and 7.3%, respectively (31). Nephrogenic systemic fibrosis related to gadolinium contrast appears to be rare, with fewer than 1,000 cases reported. Of note, this complication was limited to patients with severe renal failure with a low glomerular filtration rate (< 30 ml/min/1.73 m^2^) (75). Regarding potential device issues, MR-conditional implantable electronic devices have improved CMR compatibility with no changes in thresholds and pacemaker parameters (76). Although the impact on image quality should be considered, some studies demonstrated that patients with non-conditional devices can safely undergo the exam given proper protocols are used (77, 78).

## Conclusion

Despite some discussion to the contrary, the ISCHEMIA trial provides several crucial findings regarding the contemporary management of CAD and the clinical impact of coronary revascularization. In accordance with current guidelines (2, 3), both conservative and invasive strategies remain useful in the management of patients with CAD.

Among the non-invasive stress methods, stress CMR is recognized as an accurate technique to detect inducible myocardial ischemia and infarction with high sensitivity and specificity. Moreover, several large studies have shown its excellent prognostic value for predicting CV events. Recently, a first-line stress CMR-based strategy was shown to be non-inferior in terms of outcomes compared to an invasive approach with FFR in patients with stable angina. Given that stress CMR was used in only 5% of the patients from the ISCHEMIA trial, we may wonder about the results of a new randomized controlled trial including patients with severe ischemia defined only by stress CMR. The use of the optimal medical treatment in asymptomatic or mildly symptomatic patients who fit the profile of the ISCHEMIA trial (5) may be the best initial strategy without the benefit of coronary revascularization. However, in symptomatic patients with frequent angina episodes, or in patients with severe ischemia, an invasive strategy may be a reasonable complementary approach to the optimal medical treatment for effective angina relief. Indeed, the ischemic burden quantified with imaging modalities is crucial for guiding coronary revascularisation and improve the cardiovascular risk stratification.

## Author Contributions

TP, MJ-H, and OC-F: made substantial contributions to conception and design, acquisition of data, or analysis and interpretation of data, and drafted the article. TP, LS, AB, AT, MJ-H, and OC-F: reviewed it critically for important intellectual content and given final approval of the version to be published. All authors contributed to the article and approved the submitted version.

## Conflict of Interest

The authors declare that the research was conducted in the absence of any commercial or financial relationships that could be construed as a potential conflict of interest.
